# Molecular-Genetic Mapping of Zebrafish Mutants with Variable Phenotypic Penetrance

**DOI:** 10.1371/journal.pone.0026510

**Published:** 2011-10-19

**Authors:** Roshan A. Jain, Marc A. Wolman, Lauren A. Schmidt, Harold A. Burgess, Michael Granato

**Affiliations:** Department of Cell and Developmental Biology, University of Pennsylvania School of Medicine, Philadelphia, Pennsylvania, United States of America; Inserm U869, France

## Abstract

Forward genetic screens in vertebrates are powerful tools to generate models relevant to human diseases, including neuropsychiatric disorders. Variability in phenotypic penetrance and expressivity is common in these disorders and behavioral mutant models, making their molecular-genetic mapping a formidable task. Using a ‘phenotyping by segregation’ strategy, we molecularly map the hypersensitive zebrafish *houdini* mutant despite its variable phenotypic penetrance, providing a generally applicable strategy to map zebrafish mutants with subtle phenotypes.

## Introduction

Subtle phenotypic gradation between individuals is common for a wide variety of traits with complex genetic and environmental regulation, including growth rate, organ size, disease susceptibility, and behavior. Many neuropsychiatric disorders present a variety of different sensorimotor symptoms with extensive variability in severity (expressivity) and penetrance within the affected populations [1]. For example, defects in startle response modulation, including sensorimotor gating, habituation, and sensitivity/responsiveness to stimulation are frequently described in many psychiatric disorders with genetic components, including anxiety disorders, ADHD, schizophrenia, and autism spectrum disorders [2], [3], [4], [5], [6], [7], [8]. However, there is often significant variation in behavioral performance within and between individuals of affected and unaffected populations, with the performance indices of control individuals frequently falling within the range of affected individuals, and vice versa [9]. This issue poses a considerable challenge in genetically identifying the causative factors of these disorders.

**Figure 1:**
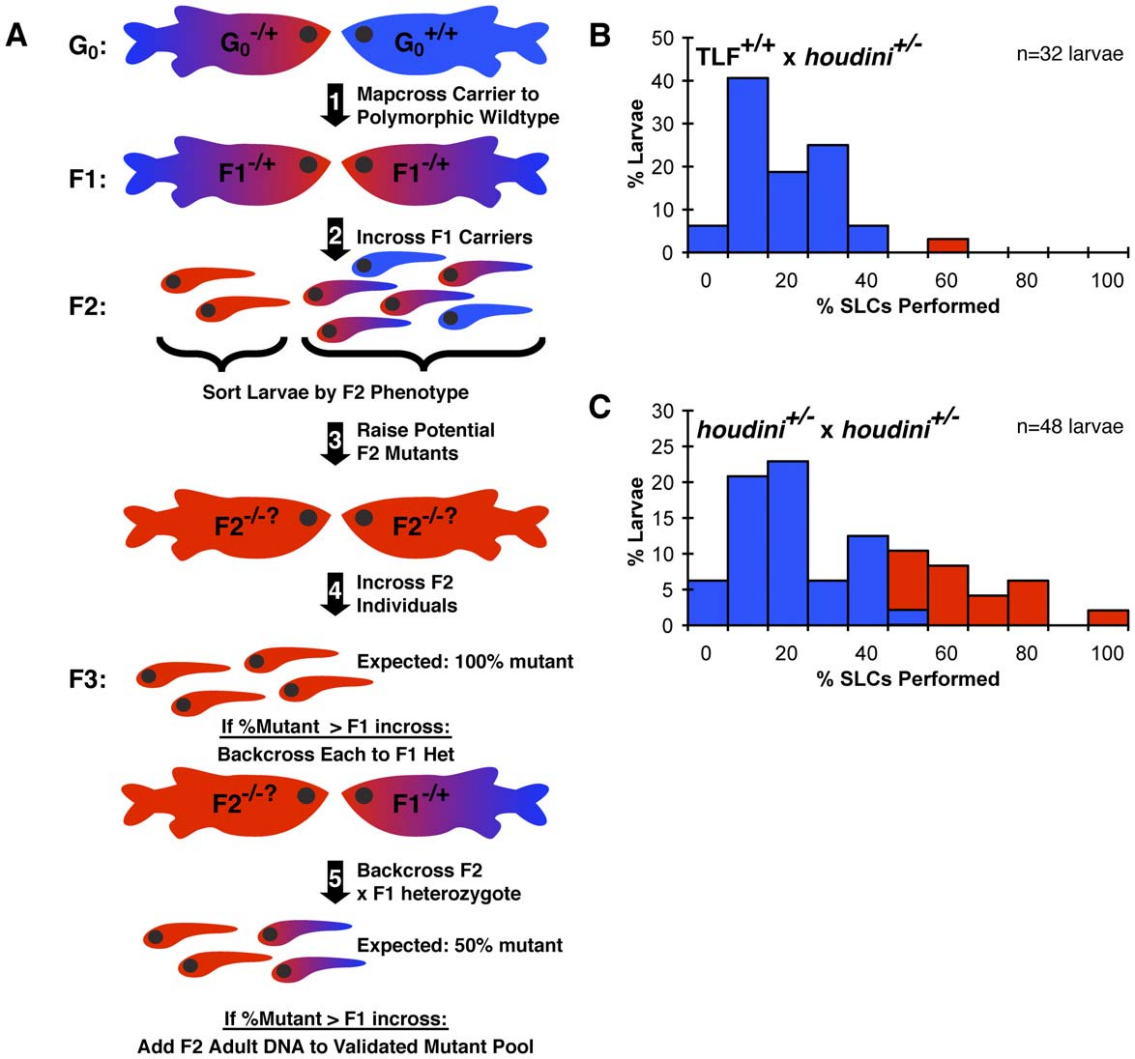
A ‘phenotyping by segregation’ strategy to map the variably penetrant *houdini* mutation. (A) Mapping zebrafish mutants with weak or variable penetrance using a ‘phenotyping by segregation’ strategy, where F3 phenotypic segregation is used to validate homozygous F2 mutants. 1) A standard mapcross is generated using a mutant carrier G_0_ and a polymorphic wildtype G_0_ [35]. 2) Heterozygous carrier F1s are isolated and incrossed to generate F2 larvae. 3) F2 larvae at the top 15% of the phenotypic range of the clutch are raised to adulthood as potential mutants, alongside an equal number of siblings (from the bottom 15% of the phenotypic range of the clutch) as controls. 4) Genomic DNA is taken from each raised F2 individual, and F2s are then randomly incrossed. 5) F2 pairs producing clutches with a greater frequency of phenotypic outliers than a control F1 heterozygous incross are next individually backcrossed to a known F1 heterozygote. Any raised F2 individual which again produced a clutch with a greater frequency of phenotypic outliers than the control F1 heterozygote incross is deemed a “validated” mutant, and is used for subsequent bulked segregant mapping. (B-C) Distributions of SLC startle responsiveness to weak subthreshold acoustic stimuli in 5 dpf larval progeny of a *houdini* heterozygote and a wildtype TLF adult (B) and two heterozygous *houdini* carriers in the same genetic background (C). Responsiveness was measured over 20 weak “subthreshold” acoustic stimuli. The mean %SLC+2SD was set as the hypersensitivity threshold for each experiment, 42% in this example. If >15% of a clutch performed above the hypersensitivity threshold for the experiment (in red), both parents were considered to carry the recessive *houdini* mutation.

Zebrafish are rapidly proving to be an excellent model system in which to genetically dissect a wide variety of motor and cognitive behaviors and disease endophenotypes [10], [11], [12], [13]. Unbiased forward genetic screens for behavioral mutants have been successfully performed, and an extensive array of mutants have been isolated and cloned via their neuromorphological defects during development [14], [15], [16], [17], [18]. However, mapping and molecularly identifying mutants purely based on their behavioral phenotype in the absence of a visible anatomical defect has been much more challenging, and relatively few have been mapped and cloned in this fashion [17], [19], [20], [21], [22], [23]. To identify genetic factors regulating acoustic startle responsiveness that may be relevant to neuropsychiatric disorders, we previously reported a forward genetic screen of ENU-mutagenized zebrafish larvae at 5 days post-fertilization (5 dpf) for mutants with subtle defects in the sensitivity and gating of the larval acoustic startle response [18]. Many of these mutants, including the hypersensitive mutant *houdini*, were morphologically normal and initial attempts at standard bulked segregant mapping failed, likely since the overlapping phenotypic variance of mutant and wildtype individuals led to misclassification of siblings as mutants (see below). To overcome this misclassification at the larval stage, we adopted a ‘phenotyping by segregation’ strategy to map the *houdini* mutant, broadly applicable to mutants with variable phenotypic expressivity and penetrance (Figure 1A).

## Results

### ‘Phenotyping By Segregation’ To Verify Mutants For Molecular Mapping

In standard bulked segregant mapping, F2 individuals from a polymorphic mapping cross are pooled based on phenotype, then analyzed for phenotypic linkage to a chromosomal region [24]. However, significant phenotypic overlap between mutant and sibling populations will result in misclassification of wildtype siblings as mutants, occluding linkage of the mutant pool. Therefore, to confirm that phenotypic outliers at the larval stage are indeed homozygous mutant individuals, we raised putative mutant individuals and their wildtype siblings to adulthood, then test crossed these individuals and examined the phenotypic ratios of their larval offspring (Figure 1A). Those putative F2 mutant adults producing F3 progeny in the ratios expected for homozygous mutant individuals would then be considered “validated” mutants and used for mapping. Thus a “validated” mutant must both exhibit the mutant larval phenotype, and produce progeny in expected Mendelian phenotypic ratios.

To verify the effectiveness of the ‘phenotyping by segregation’ strategy, we used it to map the novel behavioral mutant *houdini* isolated during our previously described startle modulation screen [18]. Like all vertebrates, zebrafish exhibit a robust and rapid startle response following sudden acoustic stimuli, and the frequency of eliciting this stereotyped response decreases as stimulus intensity decreases [18], [25], [26], [27]. *houdini* mutant larvae are hyperresponsive to weak, or “subthreshold,” acoustic stimuli relative to their wildtype siblings who respond to less than 50% of stimuli at this level (Figure 1B-C). Despite this apparent hypersensitivity, *houdini* mutants appear morphologically normal and the startle responses performed following acoustic stimuli (Short-Latency C-bends, or SLCs) are kinematically indistinguishable from wildtype SLCs (Figure S1). To distinguish *houdini* mutants from siblings, we established a subthreshold acoustic stimulus intensity eliciting a mean SLC responsiveness of 16±13% (mean ± SD) in wildtype larvae, then set a responsiveness threshold at >2 SD's above the mean, classifying individuals performing above this threshold as hypersensitive (Figure 1B). By this criterion, 15–30% of larvae from *houdini* carrier incrosses were hypersensitive, consistent with the expected frequency of 25% for a single recessive mutation (Figure 1C). To demonstrate the heritability of the *houdini* phenotype, we outcrossed *houdini* carriers to wildtype (TLF) fish. These offspring were raised to adulthood, incrossed at random, and clutches were analyzed for the hypersensitive *houdini* phenotype. Consistent with Mendelian inheritance of a single recessive mutation, 8/32 crosses again produced clutches with 15–30% hypersensitive larvae (data not shown).

**Figure 2:**
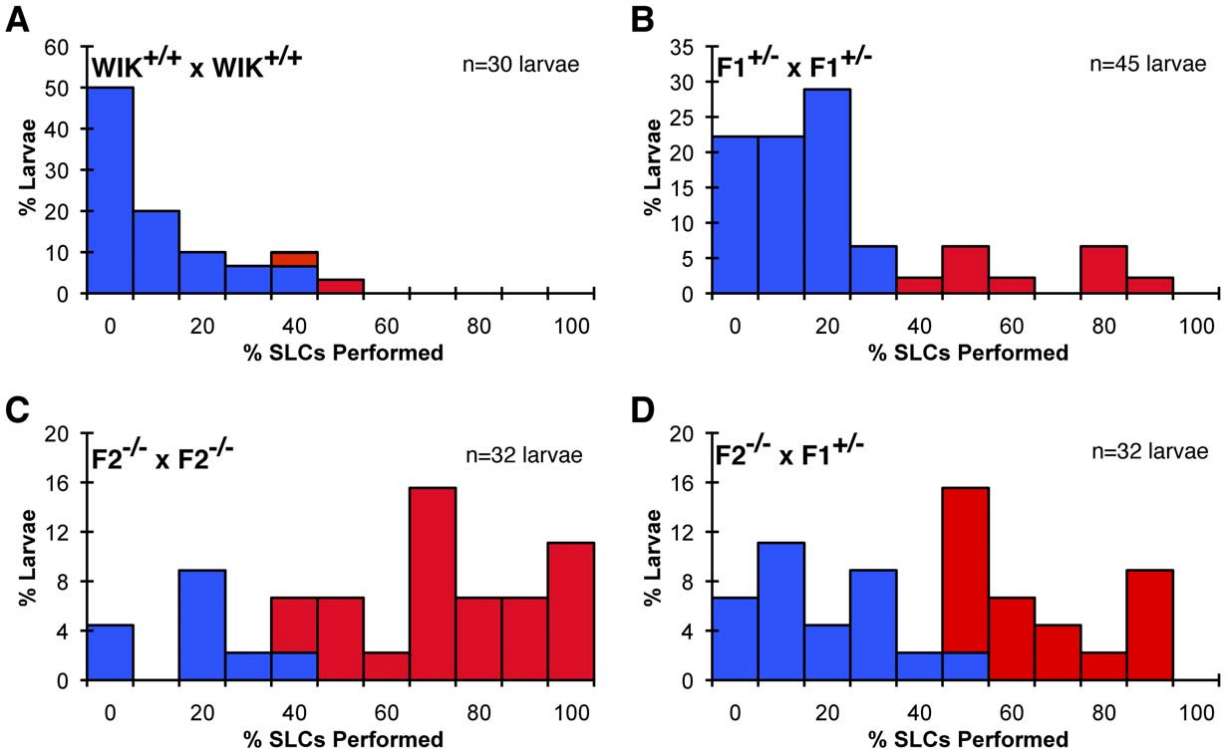
Identification of homozygous *houdini* mutants using ‘phenotyping by segregation.’ (A-D) Representative distributions larval responsiveness within F2 and F3 clutches, with hypersensitive larvae displayed in red. Larval behavior was stimulated and analyzed as in Figure 1. (A) A wildtype (WIK) incross produced larvae with a meannSLC responsiveness of 8.9±13.5% (mean±SD). The hypersensitivity threshold was set at 36% SLC responsiveness (mean + 2SD), where 2/30 (6.7%) wildtype larvae were classified as hypersensitive. (B) A F1 *houdini* heterozygous incross produced 9/45 (20%) hypersensitive larvae. (C) An incross of raised *houdini* F2 mutants produced 24/32 (75%) hypersensitive larvae. (D) A backcross of a raised F2 mutant from (C) and a known F1 heterozygote produced 16/32 (50%) hypersensitive larvae. This cross was performed on a separate occasion where the hypersensitivity threshold was set to 43% based on the wildtype (WIK) control.

To map the *houdini* mutation to a chromosomal region, we crossed a *houdini* carrier to a polymorphic wildtype strain (WIK), classified F2 larvae as putative mutants or wildtype siblings based on startle sensitivity at 5 dpf, then raised mutant and wildtype sibling F2 larvae separately to adulthood (Figure 1A) [28]. To evaluate homozygosity of the adult F2s, potential mutant F2 individuals were first incrossed, and the distribution of F3 larval responsiveness was compared to known wildtype and F1 heterozygous incross clutches. Second, potential adult F2 mutants were backcrossed to known F1 heterozygotes, again comparing the larval responsiveness distribution to wildtype and F1 heterozygous incrosses. Theoretically, crosses of two *houdini* homozygotes should produce 100% hypersensitive F3 progeny, a homozygote and a heterozygote should produce 50% hyper-responsive progeny, and two heterozygotes should produce 25% hypersensitive progeny. However, given the variable penetrance of the *houdini* phenotype, we expected that even a clutch of 100% *houdini* homozygous larvae would still show a responsiveness distribution partially overlapping the normal range of wildtype responsiveness. Therefore, we always incrossed potential F2 *houdini* homozygotes alongside F1×F1 heterozygous crosses and F1×WIK outcrosses, classifying the F2 pair as potential homozygotes or heterozygotes only if their clutch contained a significantly higher fraction of hypersensitive larvae than the known F1×F1 cross (Figure 2).

In a representative experiment (Figure 2), a wildtype (WIK) incross produced a hypersensitivity threshold of 36% (wildtype mean 8.9±13.5%), such that only 2 of 30 (6.7%) wildtype larvae were declared hypersensitive (Figure 2A). A F1 heterozygous incross resulted in 9/45 (20%) hypersensitive larvae (Figure 2B). In striking contrast, a sizable majority of larvae (24/32, 75%) were hypersensitive in some incrosses of raised hypersensitive F2 fish (Figure 2C). This frequency of hypersensitivity was never observed in any wildtype or heterozygote incross clutches, suggesting one or both F2 parents were *houdini* homozygotes (Figure S2). To determine which was the case, all F2 individuals producing clutches with >35% hypersensitive larvae were backcrossed to known F1 heterozygotes, and the clutches were again compared to wildtype and heterozygous incrosses as before (Figure 2D). F2 individuals still producing clutches with >35% hypersensitive larvae were considered “validated” *houdini* homozygotes and were used for mapping. We used a >35% threshold cutoff for assessing F2 incrosses as wildtype and known F1 heterozygote incrosses reliably fell below this cutoff, thus we reasoned that this criterion should exclude from further analysis any crosses lacking at least one mutant F2 individual. Similarly, the >35% threshold for F2 backcrosses was expected to eliminate F2 heterozygotes from the pool of “validated” homozygotes (Figure S2). We note that even in crosses of verified homozygous *houdini* individuals where the entire clutch is maternal & zygotic *houdini* mutant, 25–50% of individuals still are similarly responsive to wildtype larvae (Figure 2D, data not shown), demonstrating the incomplete penetrance of the assayed behavioral phenotype. Thus by examining phenotypic segregation in the F3 generation in addition to F2 phenotypes, we could increase the stringency of phenotypic classification to overcome issues of variable phenotypic penetrance and overlapping wildtype and mutant behavioral variance when just assaying F2 larval behavior, producing a validated mutant pool for bulked segregant analysis.

### Molecular Mapping Of *houdini*

**Figure 3:**
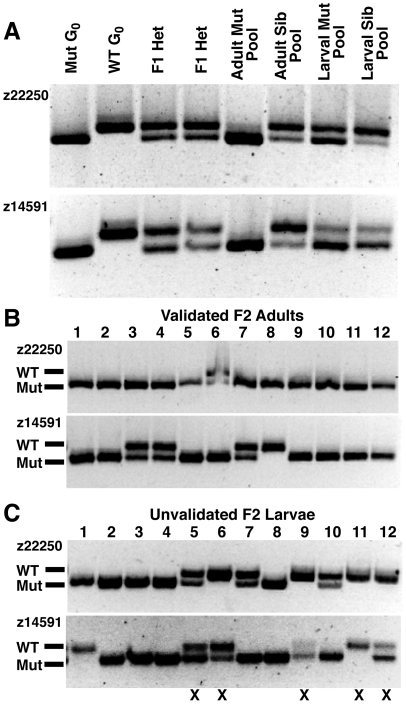
*houdini* mutants verified by F3 segregation show molecular linkage to chromosome 5. (A) The pool of F2 mutants validated by F3 segregation (“Adult Mut Pool”), shows strong linkage to the z22250 and z14591 alleles on chromosome 5 of the mutant G_0_ grandparent. Very little linkage to the G_0_ mutant alleles is evident using a pool of unvalidated mutants selected only by F2 larval behavior (“Larval Mut Pool”), or control F2 larval siblings (“Larval Sib Pool”) with these same markers. (B) Individuals composing the validated adult F2 mutant pool. 11/12 individuals are homozygous for the mutant z22250 allele, while 8/12 individuals are homozygous for the mutant z14591 allele. Individual #8 is homozygous for the mutant z22250 and wildtype z14591 alleles, indicating both the maternal and paternal copies of the chromosome underwent meiotic recombination between the *houdini* mutation and z14591. (C) 12 representative individuals from the unvalidated larval F2 mutant pool. The F2 individuals raised or pooled all performed above the hypersensitivity threshold and furthermore were among the most responsive 15% of their clutch. 5/12 larvae contain wildtype z22250 and z14591 alleles (marked with X) and are likely not homozygous mutants.

To molecularly link *houdini* to a genomic region, we used pooled DNA from 12 F2 mutants validated by the ‘phenotyping by segregation’ strategy to look for linkage to SSLP markers. The “validated” F2 mutant pool showed strong linkage to the z22250 and z14591 markers on chromosome 5, whereas both G_0_ grandparent alleles were represented in the F2 sibling pool (Figure 3A). To confirm linkage of *houdini* to these two SSLP markers, the individuals comprising the adult F2 pools were tested (Figure 3B). 11/12 individuals were homozygous for the mutant z22250 allele, while 8/12 individuals were homozygous for the mutant z14591 allele. The presence of the wildtype allele in any mutant individual indicates that individual is either a *houdini* homozygote in which meiotic recombination occurred between the wildtype and mutant chromosomes in an F1 parent, or a *houdini* heterozygote which was misclassified based on its behavior. As these two markers have been mapped as 25.5 cM apart (i.e. showing a 25.5% meiotic recombination frequency), the observed frequency of segregation of z22250 and z14591 alleles (6/24 meioses, or a 25.0% recombination frequency) is consistent with classifying these as recombination events. Importantly, the individual heterozygous at the z22250 locus (#6) was homozygous for the z14591 mutant allele, and the individuals carrying the wildtype z14591 allele (#3,4,7,8) were all homozygous for the z22250 mutant allele, suggesting that these individuals are all *houdini* mutants carrying recombinant chromosomes, rather than misclassified individuals. These data additionally suggest that these markers are on opposite sides of the *houdini* mutation. In contrast to the homozygous frequencies observed in validated F2 adult mutant individuals, only 2/49 and 9/49 F2 adult wildtype sibling individuals were homozygous for the mutant alleles of z22250 and z14591, respectively (data not shown). Furthermore, 39/49 of these sibling individuals contained wildtype alleles of both markers, indicating they are likely heterozygous or homozygous for the wildtype *houdini* locus (data not shown). As a result, the F2 adult sibling pool did not show any enrichment of the G_0_ mutant alleles for these markers, even showing a slight enrichment of the wildtype allele in the case of the z14591 marker (Figure 3A).

In contrast to the clear linkage observed between *houdini* and the markers z22250 and z14591 using a pool of “validated” adult F2 individuals, only very weak-to-no linkage is observed with respect to the G_0_ mutant alleles using a pool of “unvalidated” F2 mutant larvae (Figure 3A). This discrepancy becomes clear when the individuals comprising the “unvalidated” F2 mutant pool are analyzed with both markers. 49% (54/111) of behaviorally-isolated hypersensitive F2 larvae appear likely to be wildtype or heterozygous at the *houdini* locus (12 representative larvae shown in Figure 3C). Thus even a marker precisely at the mutant locus would not appear strongly linked using this “unvalidated” larval pool.

## Discussion

Using the ‘phenotyping by segregation’ strategy, we were able to stringently identify F2 *houdini* mutants and link the mutation to an interval on chromosome 5, indicating this approach could be broadly applicable in mapping mutants whose phenotypic spectrum significantly overlaps with that of the wildtype. Indeed, similar approaches have been exploited in other model organisms ranging from selfing plants to mice, where progeny ratios are used to guide phenotypic classification [29], [30], [31]. In considering the general applicability of this strategy, there are a number of factors which may influence the yield of “validated” mutants and overall success of the ‘phenotyping by segregation’ approach.

### Mutant Viability

The described strategy requires homozygous mutants to survive to sexual maturity, so mutations which reduce the viability or fertility of affected individuals would reduce or eliminate the frequency of homozygotes to be selected from the raised mutants, increasing the number of F2s which must be raised and tested to produce a mutant pool. However, the adult viability should not alter the penetrance of the phenotype in F3s from surviving F2 mutants. In the case of *houdini*, 46/78 (59%) of raised adult F2 individuals genotyped prior to phenotyping appear molecularly to be *houdini* homozygotes, suggesting a reduced viability of mutants is unlikely to be influencing our yield of validated mutants (p = 0.184 vs 111 behaviorally selected F2 larvae described above, Fisher's exact test, data not shown).

### Maternal Effects On Phenotype

A maternal contribution to the phenotype would predict a lower penetrance or expressivity of the phenotype of F3s from F2 heterozygous mothers than from F2 homozygous mothers. This could cause some homozygous F2 males to be excluded if they were initially tested against heterozygous females, reducing the total yield of validated mutants and skewing the ratio of validated mutants toward females as homozygous males may not reliably be identified. In the case of *houdini*, we validated 5/21 homozygous F2 females and 6/25 homozygous F2 males, arguing against a significant maternal effect influence on the mutant validation strategy in this case (p = 1.000, Fisher's exact test, data not shown).

### Degree Of Phenotypic Overlap Between Wildtype And Mutants

Many human neuropsychiatric diseases with underlying genetic factors show significant overlap in phenotypic expressivity and penetrance between mutant and non-mutant individuals, so it is likely that a similar overlap will be observed in many genetic models of these diseases. In the case of the F2 individuals tested and raised in the *houdini* mapcross described above, the most sensitive 15% of the clutch contained 59% *houdini* homozygotes and 41% heterozygous or wildtype siblings, whereas the least sensitive 15% of the clutch only contained 4% *houdini* homozygotes and 96% heterozygous or wildtype siblings (data not shown). These data indicate that while *houdini* mutants are clearly skewed toward hypersensitive performance in this assay, there is still a significant overlap in performance with that of wildtype individuals. Nonetheless, the approach described was still able to reliably exclude siblings from the mutant analysis.

### Stringency Of Selection Criteria

Clearly for the described strategy to be effective, an appropriate threshold should be set when analyzing F2 incrosses and test crosses. Too lenient of a threshold will allow nonmutant individuals into the mapping pool, while too stringent of a threshold will significantly increase the workload required to generate a mapping pool. In applying this strategy to *houdini*, we established the threshold empirically following analysis of the first 2 experimental days of testing raised F2 incrosses (Figure S2). We set a minimum threshold of 35% hypersensitivity/clutch which just excluded all previous tested crosses of known *houdini* heterozygotes, yet some F2 incrosses reproducibly produced F3 phenotype ratios exceeding this bar. Using our selection criteria on raised F2 individuals, we validated 12 of the 46 raised F2 *houdini* homozygotes (26%), thus enriching the frequency of homozygous *houdini* mutants in our raised F2 mutant pool from 46/78 (59%) to 12/12 (100%).

Having used the “validated” mutant pool to map *houdini* to a genomic interval, the next step will be to identify the mutated gene through fine recombinant mapping and sequencing approaches. To this end, we collected and stored additional putative F2 mutant larvae while waiting for mutant F2 fish to mature for segregation analysis. Although these larvae are likely to contain a significant number of non-mutant individuals, most of these can be genetically identified and discarded using the linked markers flanking the mutant locus (Figure 3C). As it is rare to observe multiple recombination events on a chromosomal region [32], any individuals heterozygous or homozygous for the wildtype marker alleles on both sides of the mutation are likely to be missorted individuals rather than recombinants, and they can be discarded. By collecting individual larvae for fine mapping during the F2 maturation interval prior to the described segregation analysis, we made maximal use of the established mapping cross and avoided potential limitations in F2 yield due to aging or death of F1 heterozygotes.

In sum, we were able to successfully map the *houdini* mutation to a chromosomal region using mutants validated by a ‘phenotyping by segregation’ approach where mapping had failed with unvalidated larvae due to the variable penetrance of the *houdini* phenotype, demonstrating the effectiveness of this approach. Thus ‘phenotyping by segregation’ analysis is likely to be a generally effective strategy for mapping viable mutations affecting traits exhibiting a phenotypic spectrum, such as the subtle behavioral deficits that model neuropsychiatric disorders, generated through unbiased forward genetic screens.

## Materials and Methods

### Ethics Statement

All experiments were conducted according to an Animal Protocol fully approved by the University of Pennsylvania Institutional Animal Care and Use Committee (IACUC) on 4–06–2010, protocol number 801067. Veterinary care is under the supervision of the University Laboratory Animal Resources (ULAR) of the University of Pennsylvania.

### Zebrafish Strains Used

Wildtype strains used were Tupfel Long-Fin (TLF) or WIK-L11 (WIK), as specified in the text [28]. The *houdini* mutation was generated by ENU mutagenesis in a mixed AB/Tü background as previously described, then outcrossed several generations to wildtype TLF individuals prior to mapping [18]. For the described mapping cross, the initial G_0_ *houdini* carrier was male, and all subsequent crosses were performed reciprocally with respect to sex. No consistent maternal or paternal effects were observed on the larval phenotype (see Discussion). Due to the excessive escape responses of mutant larvae, the *houdini* mutation was named after the famed escape artist Harry Houdini.

### Analysis Of Larval Behavior

5 dpf larval zebrafish were raised at 28°C at a density of 20 larvae/9 mL E3 embryo media, and were tested for acoustic startle sensitivity as previously described [27]. Briefly, the testing arena consisted of a 4×4 clear plexiglass grid of 16 0.9×0.9 cm chambers mounted in a 6 cm petri lid resting on a metal ring which transmitted the stimuli from a vibration exciter (4810; Brüel and Kjaer, Norcross, GA), controlled by an digital–analog card (PCI-6221; National Instruments, Austin, TX). The arena was diffusely illuminated for imaging from below with a 96-bulb infrared LED array (IR100 Illuminator removed from its housing; YYtrade) and obliquely from above with a white light LED bulb (PAR38 LED light; LEDlight.com). Each of the 16 chambers was filled with 200 µl E3 embryo media and 1 larva, and all larval responses were recorded using a high speed camera (Motionpro 2000; Redlake, Tucson, AZ) at 1000 fps with 512×512 resolution.

Two or three sets of 16 5 dpf larvae per clutch were given 20 weak “subthreshold” acoustic stimuli (5–20 m/sec^2^ waveforms of 1000 Hz) of 2 msec in duration with an interstimulus interval of 20 seconds as previously described [27]. The precise acoustic stimulus intensity was set and verified for each experimental day to produce a mean SLC responsiveness of 5–20% in wildtype 5 dpf larvae, and the mean %SLC+2SD of the wildtype larvae was set as the hypersensitivity threshold for each experiment. This helped control for any slight inter-experimental variations in environmental or handling conditions that might affect observed responsiveness. Each larva was recorded for 30 msec before and 90 msec after each stimulus, remaining isolated in individual 0.9×0.9 cm chambers throughout the experiment so that the responses of each larva could be followed across all stimuli. Automated behavioral analysis was performed using FLOTE software to identify SLC maneuvers by their robust and stereotyped kinematic parameters [18], [22], [27], [33].

### Molecular Mapping

Genomic DNA was isolated from individual larvae or tail fin clips, and larval pools were generated from equal amounts of DNA from each of 24 phenotypically mutant or sibling larvae. Adult F2 pools were similarly composed of DNA from 12 “validated” F2 adult homozygous mutants or siblings, verified by F3 segregation. Initial bulked segregant mapping of adult mutant and sibling pools was performed using a set of 168 SSLP markers spaced every 10–30 cM across all 25 zebrafish chromosomes. Linkage of the mutant pool to markers was confirmed by testing the individuals composing the pool. The two markers linked to the *houdini* mutation have both been mapped to chromosome 5 of the zebrafish genome, at 5∶67,609,750 (z22250; GenBank:G40304) and 5∶74,225,799 (z14591; GenBank:G46733) of the Zv9 Ensembl assembly (release 61; http://www.sanger.ac.uk/Projects/D_rerio/), defining a 6.6 Mb interval. The markers have additionally been mapped to positions 72.7cM (z22250) and 98.2 cM (z14591) on linkage group 5 of the MGH zebrafish linkage map [34].

## Supporting Information

Figure S1 **Startle response kinematics are unaffected in** ***houdini*** **mutant larvae.** 5 dpf larval progeny of *houdini* heterozygous parents were tested for hypersensitivity with 20 “subthreshold” acoustic stimuli as described in Figure 1. Hypersensitive larvae responding above the mean+2SD hypersensitivity threshold of wildtype controls were designated *houdini* larvae (red, n = 14), and the remaining were grouped as siblings (blue, n = 30). (A) The latency to startle initiation (“Latency”) and duration of the initial C-bend (“Duration”) were not significantly different between hypersensitive and sibling larvae. (B) The maximal turning angles (“Max Turn Angle”) and maximal body curvatures (“Max Curvature”) achieved during the initial C-bend were also not significantly different between hypersensitive and sibling larvae.
(TIF)

Figure S2 **Frequencies of hypersensitive larvae in** ***houdini*** **phenotyping crosses.** A representative set of hypersensitivity frequencies observed in the 5 dpf larvae of clutches from the *houdini* phenotyping crosses detailed in Figure 2. Crosses analyzed were: incrosses of the WIK wildtype mapping strain (WIK WT × WT, n = 6), incrosses of known F1 *houdini* heterozygotes (F1 Het × Het, n = 15), incrosses of raised F2s that were hypersensitive as larvae (F2 Mut × Mut, n = 50), incrosses of raised F2s that showed normal sensitivity as larvae (F2 Sib × Sib, n = 36), backcrosses of raised hypersensitive F2s with known F1 *houdini* heterozygotes (F2 Mut × Het, n = 8), and backcrosses of raised sibling F2s with known F1 *houdini* heterozygotes (F2 Sib × Het, n = 15). 28–32 larvae were tested in each clutch analyzed. The frequencies of hypersensitive larvae were calculated using the mean+2SD hypersensitivity threshold for each testing date, as described in the text. Based on these data, a cutoff of 35% hypersensitivity was set (red dashed line) to classify F2 incrosses. One or both F2 parents of clutches exceeding this cutoff were deemed likely to be homozygous *houdini* mutant F2s, and only these individuals were backcrossed to F1s. Data were collected across 4 weeks of testing and if parents were crossed multiple times during that period, each clutch was analyzed and graphed independently.
(TIF)
