# Supplementary figures and images for: Molecular-Genetic Mapping of Zebrafish Mutants with Variable Phenotypic Penetrance
Source: PLoS One. 2011 Oct 19;6(10):e26510. doi: 10.1371/journal.pone.0026510 (PMC3198425; doi:10.1371/journal.pone.0026510)

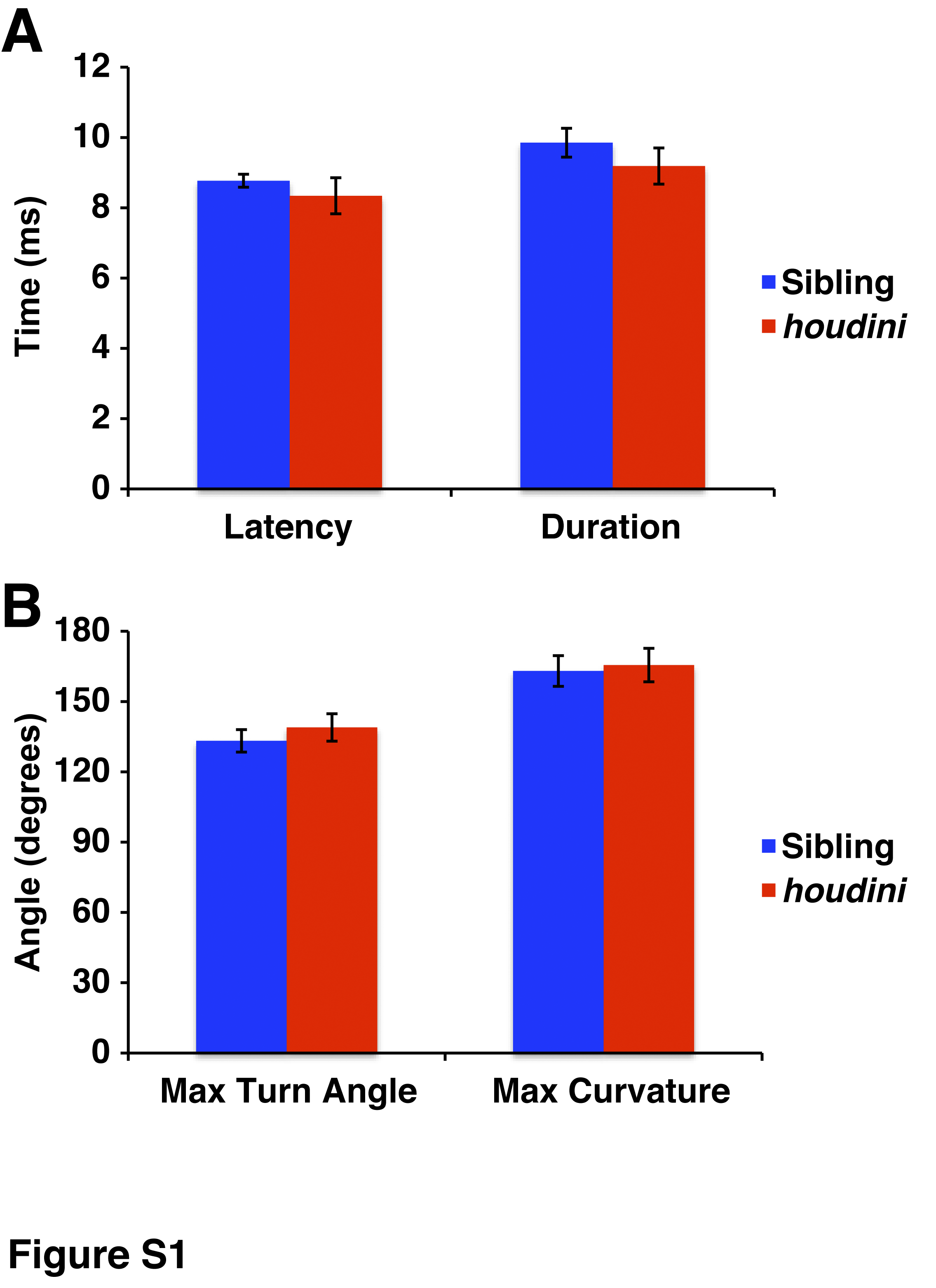

Supplement: Figure S1 — Startle response kinematics are unaffected in houdini mutant larvae. 5 dpf larval progeny of houdini heterozygous parents were tested for hypersensitivity with 20 “subthreshold” acoustic stimuli as described in Figure 1. Hypersensitive larvae responding above the mean+2SD hypersensitivity threshold of wildtype controls were designated houdini larvae (red, n = 14), and the remaining were grouped as siblings (blue, n = 30). (A) The latency to startle initiation (“Latency”) and duration of the initial C-bend (“Duration”) were not significantly different between hypersensitive and sibling larvae. (B) The maximal turning angles (“Max Turn Angle”) and maximal body curvatures (“Max Curvature”) achieved during the initial C-bend were also not significantly different between hypersensitive and sibling larvae. (TIF) [file pone.0026510.s001.tif]

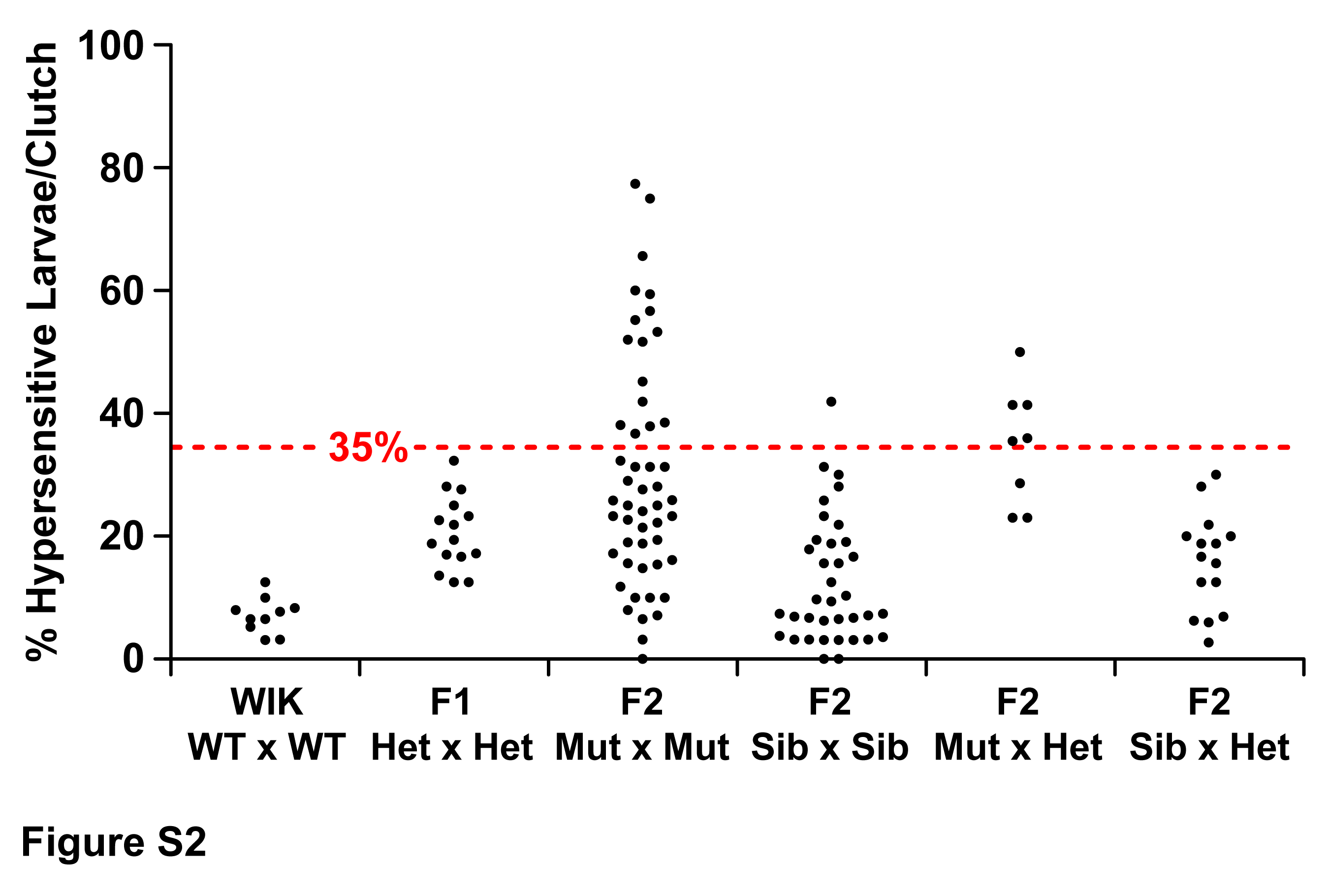

Supplement: Figure S2 — Frequencies of hypersensitive larvae in houdini phenotyping crosses. A representative set of hypersensitivity frequencies observed in the 5 dpf larvae of clutches from the houdini phenotyping crosses detailed in Figure 2. Crosses analyzed were: incrosses of the WIK wildtype mapping strain (WIK WT × WT, n = 6), incrosses of known F1 houdini heterozygotes (F1 Het × Het, n = 15), incrosses of raised F2s that were hypersensitive as larvae (F2 Mut × Mut, n = 50), incrosses of raised F2s that showed normal sensitivity as larvae (F2 Sib × Sib, n = 36), backcrosses of raised hypersensitive F2s with known F1 houdini heterozygotes (F2 Mut × Het, n = 8), and backcrosses of raised sibling F2s with known F1 houdini heterozygotes (F2 Sib × Het, n = 15). 28–32 larvae were tested in each clutch analyzed. The frequencies of hypersensitive larvae were calculated using the mean+2SD hypersensitivity threshold for each testing date, as described in the text. Based on these data, a cutoff of 35% hypersensitivity was set (red dashed line) to classify F2 incrosses. One or both F2 parents of clutches exceeding this cutoff were deemed likely to be homozygous houdini mutant F2s, and only these individuals were backcrossed to F1s. Data were collected across 4 weeks of testing and if parents were crossed multiple times during that period, each clutch was analyzed and graphed independently. (TIF) [file pone.0026510.s002.tif]
